# 

**Published:** 1915-02-13

**Authors:** 


### Buildings Contemplated.

Amersham.—The Local Government Board have held an inquiry into an application of the Rural District Council for sanction to a loan for the provision of an infectious diseases hospital.

Bristol.—The Guardians have resolved to apply to the Local Government Board for sanction to borrow an additional £8,000, making a total of £44,000, for the Southmead Infirmary.

Godalming.—Plans for the construction of a sanatorium at Godalming have been passed by the Metropolitan Asylums Board. The building will cost £43,000.

Lincoln.—The Corporation have granted the Dawber Charity Committee a further sum of £2,000 out of the income of the trust fund for the John Dawber Memorial, which is to take the form of a sanatorium. The total cost is estimated at £11,000.

Mansfield.—The Town Council have decided to take up a loan of £5,000 from the Public Works Loan Board for the extension of the Forest Hospital.

Mersham.—The East Ashford Rural District Council propose to build a smallpox hospital at Mersham. The plans and specifications are now under consideration.

Odsal.—The Bradford Corporation Health Committee propose to provide a temporary smallpox hospital on part of the Odsal tip lands. The scheme will be submitted for the approval of the Local Government Board.

Sheffield.—The extensions to the Lodge Moor Hospital are to be carried out in three sections. In the first year the work will comprise the extension of the day nurses' home, the extension of the maids' home, a stores block, alterations to the administration buildings, a lecture theatre, discharge pavilion, etc., at a total approximate cost of £10,000; in the second year, three central ward blocks, with connecting corridors, reception pavilions, and workshop and destructor-house, etc., will be built at a cost of £20,000; and in the third year the contract, to the amount of £33,500, will comprise the north three ward blocks, a night nurses' home, a private patients' block, eight cottages, etc.

### Medical Appointments.

Mr. Thomas John Nicholl has been appointed to be fifth assistant medical officer at Bexley Mental Hospital.

Mr. O. P. N. Pearn has been promoted from third to be second assistant medical officer at Horton Mental Hospital.

The Council of the University of Sheffield has appointed Mr. Arthur J. Hall, M.A., M.D., F.R.C.P., to the post of Lecturer in Medicine.

Mr. G. E. Thornton and Mr. M. Z. Hanafy have been appointed assistant medical officers in the infectious hospitals services of the Metropolitan Asylums Board.

Dr. M. G. Paul, who graduated M.B. at Queen's University, Belfast, in January, has been appointed resident surgeon of the Children's Hospital, Dublin.

The Local Government Board has sanctioned the appointment of Dr. R. P. Marshall as temporary medical officer at Parish Street Institution, Bermondsey, at a salary of £100 per annum; at Tanner Street Institution, £150 per annum; and as temporary medical officer for No. 1 district at a salary of £160 per annum; and of Dr. V. A. Jaynes as temporary medical officer for Nos. 5 and 6 districts at a salary of £190 per annum.

Mr. George William FitzHenry, M.R.C.S. Eng., L.S.A. Lond., has been appointed by the Camberwell Board of Guardians medical officer to the East Dulwich district and public vaccinator to the Dulwich district at a salary of £105 per annum, with extra fees for midwifery, etc. Dr. FitzHenry is at present in private practice in Dulwich, and held the appointment of temporary medical officer in the district. He was previously in private practice in New Zealand, where he was visiting surgeon at H.M. prison at Lyttelton, and at various times a ship's surgeon.

### Gifts and Bequests.

The King recently sent a gift of pheasants from Windsor Great Park for the sick and wounded soldiers in the Torbay Hospital, Torquay.

The Right Hon. Robert Nathaniel Cecil George, fifteenth Baron Zouche of Haryngworth (a creation of 1308), of Parham Park, Pulborough, Sussex, who left unsettled property of the gross value of £96,218, has bequeathed £200 to Guy's Hospital and £100 to the British Home and Hospital for Incurables.

Mr. William Prenton, of Walton, Liverpool, pawnbroker, who left estate valued at £20,905 gross, has bequeathed £500 each to the Stanley Hospital, Liverpool; the Royal Infirmary, Liverpool; the Cancer and Skin Hospital, Liverpool; the Eye and Ear Hospital, Liverpool; the Northern Hospital, Liverpool; and the Southern Hospital, Liverpool.

### DOCTORS' WILLS AND BEQUESTS.

Dr. Henry Charles Hilliard, M.D., aged seventy, of Bletchingley, Surrey, formerly demonstrator of anatomy at Queen's College, Birmingham, has left estate valued at £10,587.

Dr. Thomas Maccall, M.D., aged sixty-three, of Portland Street, Southport, Lancs, has left reversionary bequests to Edinburgh hospitals to the extent of £10,332.

### Personalia.

Mr. W. J. Wormwell has been re-elected chairman of the Coventry and Warwickshire Hospital.

Mr. F. T. H. Wood, one of the London County Council's assistant medical officers, has resigned his post, having obtained another appointment.

### The Book Market.

LIST OF NEW BOOKS RECEIVED FROM THE FOLLOWING PUBLISHERS:—

J. and A Churchill: "The Vicious Circles of Neurasthenia and their Treatment." By J. B. Hurry, M.A., M.D. 3s. 6d. net.

T. Werner Laurie, Ltd.: "The Soldier's English-German Conversation Book." 7d. net. "Thou art the Man!" By Sidney Dark. *Daily Express* War Book. 1s. net. "Love Letters of a Soldier." By May Aldington. 1s. net.

Mather and Crowther, Ltd.: "Practical Advertising, 1915." 3s. 6d.

Ponsonby and Gibbs, Dublin: "Nursing Ethics." By T. Percy Kirkpatrick, M.D. 1s.

G. P. Putnam's Sons: "A Medical Dictionary for Nurses." By Amy E. Pope. 3s. 6d. net.

J. Wright and Sons, Ltd.: "Evolution and Disease." By J. T. C. Nash, M.D. 3s. 6d. net.

### Institutional and Public Reports.

Archives of the Middlesex Hospital: Clinical Series No. XIV. (being the thirty-second volume of the series). Edited by W. Sampson Handley and Victor Bonhey.

Archives of the Middlesex Hospital. Vol. XXXIII. Thirteenth Report from the Cancer Research Laboratories.

Arbroath Infirmary: Seventieth Annual Report, 1914.

### THE PAIGNTON WAR HOSPITAL.

The American Women's War Hospital at Paignton is being enlarged by thirty beds, bringing the total capacity of the hospital up to 230. This addition has been made possible by utilising the riding school. The new ward will be called the "St. George" Ward, after a generous donor to the fund, and when completed will be staffed by English and American nurses, as are all the other wards in the hospital.

### BRIDGE FOR CHARITY.

On Wednesday last week, at the Empress Rooms, Kensington, a bridge tournament was organised by Mrs. Brand and Mrs. Toms for the benefit of the Samaritan Free Hospital for Women, Marylebone Road, N.W. About sixty-five tables were arranged, and some twenty prizes were kindly presented by leading West-End firms. The greater part of the expenses being defrayed by the organisers, the function resulted in a contribution of £62 towards a much-needed electric lift for the hospital. After a vote of thanks to the organisers had been proposed, on behalf of the hospital, by Dr. C. Hubert Roberts, the prizes were distributed by Lady Henry Buckingham.

The directors of the London County and Westminster Bank, Ltd., after making provision for bad and doubtful debts, and applying £336,600 in writing down investments, have declared a dividend of $10\frac{5}{8}$ per cent. for the past half-year (less income tax), making a total distribution of $21\frac{1}{4}$ per cent. for the year 1914, leaving a balance of £160,112 to be carried forward.

RATES OF SUBSCRIPTION: Twelve months, 6/6; Six months, 4/-; Three months, 2/-, post free to any address in the United Kingdom. Abroad, Twelve months, 8/8; Six months, 5/-; Three months, 2/6, post free.—Address The Subscription Dept., "THE HOSPITAL," The Hospital Building, 28/29 Southampton Street, Strand, London, W.C.

